# A Not-So-Sweet Syndrome: A Case Report of a Male Presenting With Acute Febrile Neutrophilic Dermatosis

**DOI:** 10.7759/cureus.48810

**Published:** 2023-11-14

**Authors:** Kim-Long R Nguyen, Aaron J Lacy, SueLin Hilbert, Rosanne S Naunheim

**Affiliations:** 1 Emergency Medicine, Washington University School of Medicine, St. Louis, USA

**Keywords:** corticosteroid therapy, skin biopsy, acute febrile neutrophilic dermatosis, skin lesion, dermatology, sweet syndrome

## Abstract

Physicians often encounter patients who present with a chief complaint of skin changes or lesions in both acute and primary care settings. Early initiation of appropriate treatment and pharmacotherapy in patients who present with rash is crucial to prevent decompensation, morbidity, and further downstream utilization of hospital resources. Acute febrile neutrophilic dermatosis, more commonly known as Sweet syndrome, is a rare and highly symptomatic inflammatory skin condition. Early recognition of Sweet syndrome is important as it requires specific treatment considerations and often can be a sign of an underlying pro-inflammatory condition, malignancy, or reaction to new medication that must be identified. This article discusses the presentation and management of a 50-year-old male who presented with a classic presentation of Sweet syndrome.

## Introduction

Physicians in the acute care setting often encounter patients who present with skin changes or lesions. The recognition of concerning rashes and skin changes is important for physicians to understand as mis-triage and misdiagnosis of these patients can have a range of consequences, ranging from poor patient outcomes to overutilization of hospital resources. In patients with skin changes, appropriate therapy initiation is crucial in those who are at risk of decompensation, are highly symptomatic, or may have a serious underlying medical condition manifesting as a skin finding. Acute febrile neutrophilic dermatosis, also termed Sweet syndrome, is a rare inflammatory skin condition that has been observed in association with pro-inflammatory conditions, hematologic or oncologic malignancy, and the initiation of new medications [1,2].

## Case presentation

A 50-year-old male presented to the emergency department (ED) for skin lesions. His medical history was notable for chronic obstructive pulmonary disease, hepatitis C, and end-stage renal disease on dialysis. The painful lesions appeared one week prior to his presentation, and he was initially seen at another hospital where he was treated with intravenous antibiotics. Subsequently, his lesions enlarged and spread diffusely, prompting him to represent to the ED.

**Figure 1:**
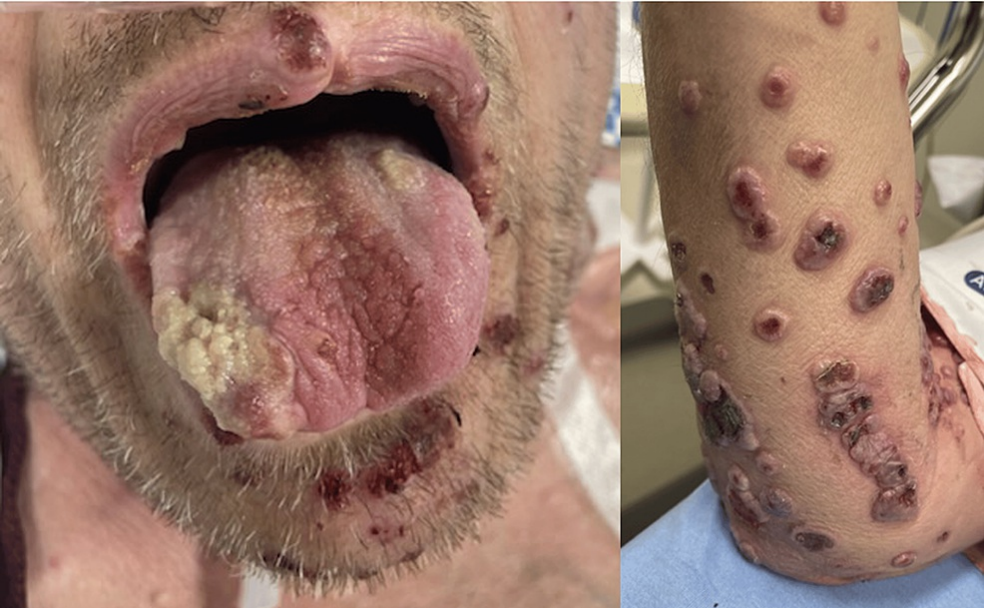
Raised, erythematous, painful, Nikolsky-negative, hemorrhagic skin lesions of the oral mucosa and extremities

**Figure 2:**
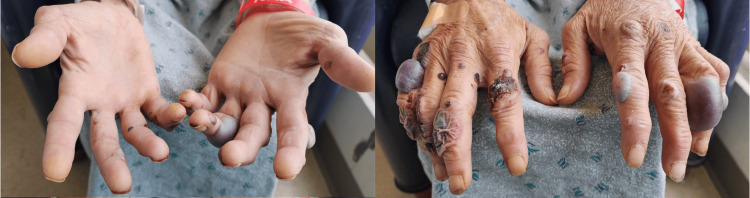
Raised, erythematous, painful, Nikolsky-negative, hemorrhagic, bullous skin lesions of the hands that spared the palms

On examination, he had normal vital signs and appeared uncomfortable. Skin examination was notable for polymorphic, raised, hemorrhagic, and bullous skin lesions with heaped margins across the body, including his intraoral mucosa (Figure 1). The lesions were Nikolsky sign negative and spared the palms and soles (Figure 2). At the time of evaluation in the ED, monkeypox, herpes simplex virus, varicella-zoster virus, blood cultures, gonorrhea/chlamydia, human immunodeficiency virus (HIV), rapid plasma reagin (RPR), and hepatitis panel testing were initiated. Ultimately, all were negative, except for a positive hepatitis C RNA result. Other notable results included a white blood cell count of 5,000/µL and a C-reactive protein of 159 mg/L. Given the concern about systemic and highly symptomatic skin changes, which could indicate concurrent infection or underlying occult disease process, he was admitted for further evaluation and empirically started on both broad-spectrum antibiotics and high-dose intravenous steroids.

**Figure 3:**
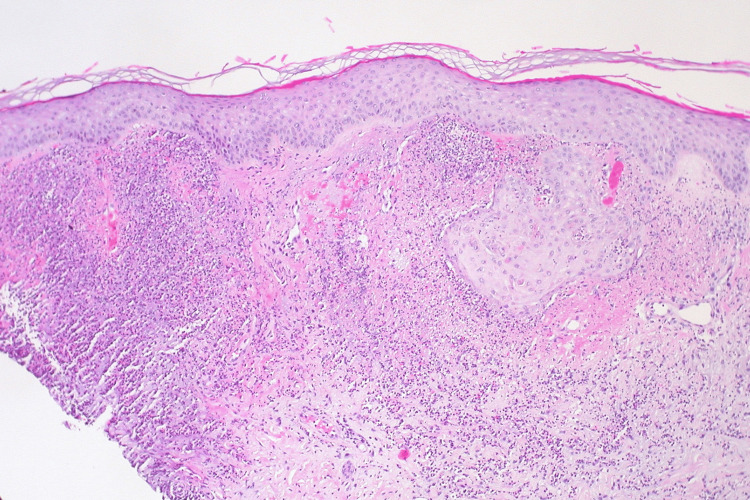
Example histopathology slide of a patient's skin biopsy showing neutrophils in the upper dermis with fibrinoid change of capillary-sized vessels consistent with a diagnosis of Sweet syndrome Figure reproduced via Creative Commons Attribution 2.0 Generic license courtesy of Ed Uthman: https://commons.wikimedia.org/wiki/File:Sweet_Syndrome_(neutrophilic_dermatosis)_(4703671002).jpg

Workup for underlying infectious, autoimmune, and oncologic disease processes by the inpatient team included cryptococcal antigens, blastomyces antibodies, histoplasma antibodies, screening computed tomography of the chest, abdomen, and pelvis, and microorganism testing of a lesion biopsy. Ultimately, these tests were negative, and skin biopsy demonstrated collections of neutrophils in the upper dermis with fibrinoid change of capillary-sized vessels, with negative microorganism testing, consistent with a diagnosis of acute febrile neutrophilic dermatosis, also termed Sweet syndrome. An example of Sweet syndrome histopathology can be seen below (Figure 3). Please note that this figure is not from this case. His antibiotics were discontinued, and treatment with 1 mg/kg of prednisone was initiated. Ultimately, after significant symptom improvement during his four-day hospital course, he was discharged with an oral prednisone taper and specialty follow-up arranged.

## Discussion

Physicians in the acute care setting often see patients who present with skin findings of undetermined origin. It is crucial for providers to recognize when a skin condition requires immediate treatment and further inpatient management or is a sign of a possible occult disease process. Misdiagnosis can lead to improper treatment initiation, increased morbidity due to delay in treatment, or failure to recognize a serious, occult disease process requiring intervention. Sweet syndrome is one such condition, as it is often associated with underlying infection or malignancy [1]. Sweet syndrome was first described in 1964 by Robert Sweet after describing eight cases of skin eruptions similar to erythema multiforme associated with fever.

The diagnosis of Sweet syndrome includes major criteria (abrupt onset of painful erythematous plaques and/or nodules, and histopathologic evidence of dense neutrophilic infiltrate without evidence of leukocytoclastic vasculitis) and two of four minor criteria (fever, known malignancy or infection, elevated inflammatory markers, and/or excellent response to systemic steroids) [1-4]. High-dose corticosteroid therapy is the first-line treatment, with topical or intralesional injections suggested for mild disease and systemic treatment for extensive/systemic disease. Colchicine, dapsone, and potassium iodide are less frequently used but also utilized [1,3]. Our patient had the rare bullous form of Sweet syndrome, which is often associated with an underlying hematologic malignancy. Fortunately, a workup for underlying malignancy was non-revealing. In this patient, early initiation of high-dose intravenous steroids ultimately resulted in a suitable outcome, with a substantial reduction in symptoms and improvement in skin findings noted on follow-up with dermatology.

## Conclusions

This is a case of a patient who presented with Sweet syndrome. Sweet syndrome often presents with both skin changes and fever, which can mimic cellulitis, prompting physicians to start empiric antibiotics. Early recognition of this disease process is important, as the treatment is steroids, not antibiotics. Importantly, providers should be aware that Sweet syndrome is commonly associated with underlying inflammatory conditions and malignancy and should prompt a thorough workup and evaluation of patients who have the disease process confirmed or suspected.
