# Correction: A Not-So-Sweet Syndrome: A Case Report of a Male Presenting With Acute Febrile Neutrophilic Dermatosis

**DOI:** 10.7759/cureus.c145

**Published:** 2023-11-21

**Authors:** Kim-Long R Nguyen, Aaron J Lacy, SueLin Hilbert, Rosanne S Naunheim

**Affiliations:** 1 Emergency Medicine, Washington University School of Medicine, St. Louis, USA

This article has been corrected to clarify that Figure 3 is not original to the case and is meant to serve only as an example of Sweet syndrome histopathology. The paragraph preceding Figure 3 has been updated to make this clear and the figure attribution and license information has been moved from the reference list to the Figure 3 description.

